# Comment on Tanmoy et al. CRISPR-Cas Diversity in Clinical *Salmonella enterica* Serovar Typhi Isolates from South Asian Countries. *Genes* 2020, *11*, 1365

**DOI:** 10.3390/genes12081142

**Published:** 2021-07-28

**Authors:** Laetitia Fabre, Elisabeth Njamkepo, François-Xavier Weill

**Affiliations:** Institut Pasteur, Unité des Bactéries Pathogènes Entériques, 75015 Paris, France; laetitia.fabre@pasteur.fr (L.F.); elisabeth.njamkepo-nguemkam@pasteur.fr (E.N.)

Tanmoy et al. [1] report new findings relating to CRISPR locus organization and composition in *Salmonella enterica* serovar Typhi (hereafter referred to as *S.* Typhi). They reported that *S.* Typhi isolates can carry up to five different CRISPR loci and about 19% of the tested genomes had three or more CRISPR loci, whereas previous studies reported only two loci [2,3], suggesting that these studies were incomplete due to the use of too small a set of *S.* Typhi genomes.

As first described by Jansen et al. [4], clustered regularly interspaced short palindromic repeats (CRISPRs) are a family of repeated DNA sequences present in prokaryotes, and they are characterized by 24–47 bp DNA direct repeats (DRs), separated by variable 21–72 bp sequences called “spacers” [5,6]. An A-T-rich “leader sequence” and *cas* (CRISPR-associated sequence) genes are often identified adjacent to the CRISPR locus. Based on the 16 to 39 assembled genomes from *Salmonella* available at the time, including two from *S.* Typhi (CT18 and Ty2), the *Salmonella* CRISPR region was characterized by our group and others as two loci, CRISPR1 and CRISPR2, separated by less than 20 kb [2,7], with type I-E *cas* genes in the interval between the CRISPR loci [8] (Figure 1).

We also investigated the polymorphism of CRISPR1 and CRISPR2 by performing a PCR (polymerase chain reaction)–Sanger sequencing analysis on 744 *Salmonella* reference strains and isolates of 130 serovars (including 18 clinical *S.* Typhi isolates). Over 3800 unique spacer sequences were identified, stored and can be queried online at: https://galaxy.pasteur.fr/root?tool_id=toolshed.pasteur.fr/repos/khillion/salmonella_crispr_typing/salmonella_crispr_typing/1.0.0 (accessed on 5 July 2021) [2]. Mean spacer length was 32 bp (29–74 bp), and a 29 bp (26–30 bp) DR consensus sequence was identified; however, single nucleotide polymorphism (SNP) variants were also observed. The strong correlation between spacer content and serovar/multilocus sequence type [2] led to a patent describing a new *Salmonella*-subtyping method [9]. In the two *S.* Typhi genomes (Ty2 and CT18) and 18 *S.* Typhi isolates from diverse genotypes (13 different haplotypes [10]), geographic origins (9 countries) and time periods (1918–2006) studied, six CRISPR1 spacer sequences (Typhi1 to Typhi6), one CRISPR2 spacer sequence (EntB0var1) and seven different combined CRISPR1/CRISPR2 profiles were identified [9]. No CRISPR1 spacer was common to all 20 *S.* Typhi genomes and isolates studied. However, a spacer, EntB0var1, was found in all CRISPR2 sequences. This spacer sequence was then used to develop a *S.* Typhi serovar-specific PCR assay, which was validated on 188 *S.* Typhi reference strains and isolates of diverse genotypes (65 different haplotypes [10]), geographic origins (40 countries) and time periods (1918–2009). This CRISPR2 target was then used in a multiplex PCR for the detection of *S.* Typhi in blood samples from Bangladesh [11].

For comparison of the results reported by Tanmoy et al. [1] with those from our previous studies [2,3], the 1059 genomes described by the authors were downloaded from EBI-ENA (https://www.ebi.ac.uk/ena/browser/home, accessed on 24 November 2020) and assembled with SPAdes [12], according to the authors’ parameters. The metrics of the assemblies (N50, genome size and N contigs) revealed evidence of the contamination of some genomes (ERR2663487, ERR2663542, ERR2663589, ERR2663887 and ERR2663969) with other *Salmonella* serovars (Enteritidis, Paratyphi A and Worthington), which was confirmed by molecular serotyping and/or multilocus sequence typing (Supplementary Materials Table S1, “Comment” column). The genotyphi program [13] was used to check the genotypes of the downloaded genomes. Intriguingly, for more than 500 of the 1059 genomes, discrepancies were observed between the results presented by the authors and our analysis (Supplementary Materials Table S2). The discrepancies related to genome sequences from a previous publication by the authors [14]. We found that the strain name/accession code pairs shown in the authors’ Dataset S1 [14] did not match those available from EBI-ENA (Supplementary Materials Table S3). We suspect that a single lane shift occurred between the accession codes and associated strain names after strain 311189_226186 (ERR2663487), either during construction of the spreadsheet or after its submission to EBI-ENA. We therefore used only the EBI-ENA accession codes as identifiers for the genomes in our review. This major issue also precluded the use of metadata, other than the country of origin (Bangladesh) and of the data published in the authors’ papers [1,14]. We analyzed the CRISPR region with both CRISPRCasFinder [15] and a script developed in-house and based on *in silico* PCR for identification of the sequences of CRISPR1, CRISPR2 and spacer/DR sequences with the SalmCRISPRtyping script [2] (Supplementary Materials Table S1).

All the *S.* Typhi genomes belonged to the type I-E CRISPR-Cas system. No new CRISPR spacer or DR sequences were identified. Eleven combined CRISPR1/CRISPR2 profiles were identified among the 1059 genomes tested (Table 1 and Supplementary Materials Table S1). The four new combined CRISPR1/CRISPR2 profiles identified since the previous analysis of 20 *S.* Typhi genomes [2,3] were due entirely to variations of the number of DR64 at the 3’-end of CRISPR1. No clear association between the combined CRISPR1/CRISPR2 profiles and genotype was observed (Table 1).

As the authors surprisingly found no correspondence between the CRISPR spacer sequences they identified and those from our previous studies (except for Ts32v), a further analysis of the sequences listed in their Dataset S3 [1] was conducted. As illustrated in Supplementary Materials Table S4, six of their most frequent “Group-A” spacers (Ts32h, Ts32c, Ts32l, Ts32e, Ts32i and Ts32g) corresponded to our previously described CRISPR1 spacers (Typhi1, Typhi2, Typhi3, Typhi4, Typhi5 and Typhi6, respectively). Another six of their “Group-A” spacers (Ts34f, Ts36a, Ts34a, Ts34b, Ts34c and Ts34e) were actually the CRISPR1 spacers described above, but with the inclusion of two to four nucleotides from the upstream or downstream DR sequences (Supplementary Materials Table S4). Seventeen of their identified *S.* Typhi “Group-A” spacers (Ts32t, Ts32u, Ts32q, Ts32s, Ts32p, Ts32k, Ts32a, Ts32d, Ts32v, Ts32f, Ts32n, Ts32w, Ts33a, Ts32b, Ts32r, Ts32o and Ts32m) actually correspond to spacer sequences from *S.* Enteritidis (Supplementary Materials Table S4). Eight of these spacers (Ts32t, Ts32u, Ts32q, Ts32s, Ts32p, Ts32k, Ts32a and Ts32d) were identified in only one genome (ERR2663969, corresponding to ERR2663968 in Reference [1]), which was a mix-up between *S.* Typhi and *S.* Enteritidis (Supplementary Materials Table S1). Surprisingly, *S.* Enteritidis spacers Ts32f, Ts32n, Ts32w, Ts33a, Ts32b, Ts32r, Ts32o and Ts32m could not be identified in any of the 1059 genomes, including ERR2663969. Regarding the DR sequences, the “Group-A” Td29a corresponded to our DR, the most frequently found direct repeat in *S.* Typhi (Supplementary Materials Table S4). We confirmed the presence of DR/Td29a in all 1059 *S.* Typhi genomes, whereas the authors did not find it in 5/1059. The frequent DR variants DR15 (SNP variant) and DR64 (26 bp long variant) we previously found in *S.* Typhi CRISPR1 were not identified by Tanmoy et al. [1]. Two of the “Group-B” DR sequences (see below), Td39a and Td29b, were the exact reverse complemented sequences of another two DRs, Td39b and Td29c, respectively [1].

We took a closer look at the different CRISPR loci to understand the discrepancies between the authors’ study and this review. First, our CRISPR2 locus was not identified by the authors in any of the 1059 genomes studied. This is not particularly surprising, as we had already reported in 2012 that CRISPRCasFinder software [15] was unable to detect the short CRISPR2 locus of *S.* Typhi strains Ty2 and CT18, which have a unique spacer (EntB0var1) between two DRs (DR27 and DR), one of which is degenerate (identity of 20/29 bp). Surprisingly, this undetected CRISPR2 target was previously used by the first and senior authors of the Tanmoy et al. [1] article, for the detection of *S.* Typhi by PCR in clinical samples [11]. The number of loci we identified (Supplementary Materials Table S1) with CRISPRCasFinder software (including loci with a low evidence score) did not really match the number of loci reported by the authors. The nucleotide sequences of each locus defined in the authors’ Table 4 were then reconstructed according to the DR-spacer sequences described in their Dataset S3 [1], and the blastn algorithm of blast+ v.2.6.0 was then used to check for these sequences. For “Group-A” loci, patterns a1 and a3 (described in their Table 4) corresponded to CRISPR1 and CRISPR2 of *S.* Enteritidis, respectively. Therefore, only patterns a2, and a4 to a7 consist of *S.* Typhi CRISPR1 spacer sequences. One of these patterns, a6, found in genomes ERR2663783 and ERR2663776, is actually identical to the 421 bp length variant of a2 and should therefore be withdrawn. In comparison, our analysis identified 11 alleles for CRISPR1 (Table 1). Some of our profiles containing a limited number of CRISPR1 spacers, such as P1 (one spacer) and P6 (two spacers), were not identified by the authors (Supplementary Materials Table S5). Our profiles P5 and P8 correspond to their patterns a4 and a7, respectively. For the other profiles, there was no good correlation with their patterns. In particular, their prevalent pattern, a2, could be broken down into eight different profiles according to our analysis, due to the presence of DR64, sometimes repeated, in various profiles (Table 1). DR64 was not identified by the authors.

Regarding the new CRISPR loci identified by the authors (corresponding to “Group-B” loci), we found that the b4, and b21/b22 patterns corresponded to the normal CRISPR1 array found downstream from the *iap* gene [2] (“group-A” locus according to Reference [1]) and not to new CRISPR loci. Pattern b21, in particular, corresponds to our profile P6 (see above). The other “Group-B” loci were scored as “low evidence” by CRISPRCasFinder (levels 0 and 1 versus level 4 for CRISPR1), and no *cas* genes were detected in their vicinity. Furthermore, most of these new CRISPR loci consist of a minimal array (DR-spacer-DR). Investigation of the most frequent “Group-B” patterns, namely b1, b9 and b10–b13 (Supplementary Materials Figure S1) identified by the authors revealed that these “Group-A” loci consisted of repeated sequences, some being genuine variable number of tandem repeats (VNTRs). Hence, in the region defining “Group-B” loci patterns b10–b13, a large sequence of 93 bp (fusion of Ts54a/b and Td39a), repeated up to six times, depending on the genome, was misinterpreted as a CRISPR locus. Consequently, single loci consisting of limited numbers of repeated sequences with no nearby CRISPR-*cas* machinery, such as the “Group-B” loci described by the authors, should not be considered to be CRISPR loci.

This analysis confirms our previous results [2,3,9] and those of other groups [7,8] by showing that the genetically homogeneous *S.* Typhi population contains a single CRISPR-*cas* system (type I-E), with two adjacent CRISPR loci. Both CRISPR loci contain a limited number of spacers (1–6 in CRISPR1 and one in CRISPR2), as observed in other host-adapted *Salmonella* serovars with altered *cas* genes [2].

## Figures and Tables

**Figure 1:**
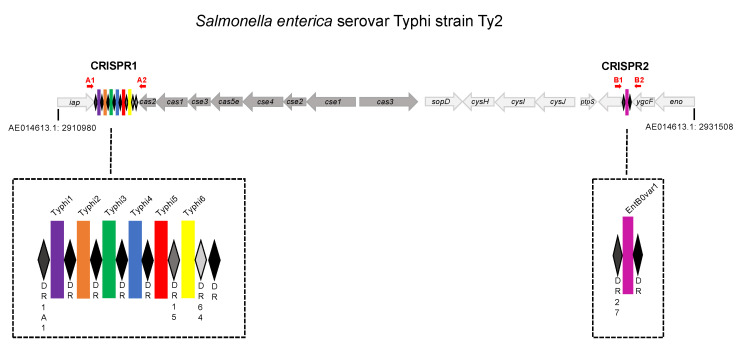
The CRISPR/Cas system structure of *S. enterica* serovar Typhi. The structure shown is that for the representative *S. enterica* serovar Typhi strain Ty2 (GenBank accession no. AE014613.1). Two CRISPR loci (CRISPR1 and CRISPR2) are present. The CRISPR-associated (*cas*) genes *cas2*, *cas1*, *cse3*, *cas5e*, *cse4*, *cse2*, *cse1* and *cas3* genes of the I-E type are located between the CRISPR loci. Diamonds represent direct repeats (DRs), with colored rectangles indicating spacers. The primers used to extract the two CRISPR loci from genomic sequences are shown as red horizontal arrows. The coordinates of the region (based on AE014613.1) are indicated.

**Table 1:** CRISPR arrays, CRISPR profiles and genotypes of the 1059 *S. enterica* serovar Typhi genomes described by Tanmoy et al. [1].

CRISPR1	CRISPR2	Combined CRISPR Profile	No. of Strains	Genotypes (n)
DR1A1-Typhi1-DR-Typhi2-DR-Typhi3-DR-Typhi4-DR-Typhi5-DR15-Typhi6-DR64-DR	DR27-EntB0var1-DR	P1	905	**4.3.1.1** (294), **4.3.1.2** (212), **4.3.1.3** (109), **4.3.1.1.P1** (88), **4.3.1** (50), **3.3.2** (47), **3.3.2.Bd2** (24), **2.0.0** (21), **2.2.0** (11), **3.3.0** (9), **2.0.1** (7), **2.1.7** (7), **3.3.1** (6), **2.3.3** (3), **3.0.0** (3), **3.0.2** (3), **ND** (3), **1.2.1** (2), **2.2.2** (2), **2.2.4** (1), **2.4.0** (1), **3.1.0** (1), **3.1.2** (1), **4.1.0** (1)
DR1A1-Typhi1-DR-Typhi2-DR-Typhi3-DR-Typhi4-DR-Typhi6-DR64-DR	DR27-EntB0var1-DR	P2	76	**3.2.2** (73), **3.2.1** (2), **3.0.0** (1)
DR1A1-Typhi1-DR-Typhi2-DR-Typhi3-DR-Typhi4-DR-Typhi5-DR15-Typhi6-DR	DR27-EntB0var1-DR	P3	40	**3.3.2.Bd1** (21), **2.5.0** (7), **2.2.0** (4), **3.3.2** (3), **2.2.2** (2), **3.0.1** (1), **3.3.0** (1), **ND** (1)
DR1A1-Typhi1-DR-Typhi2-DR-Typhi3-DR-Typhi4-DR-Typhi5-DR15-Typhi6-DR64-DR64-DR	DR27-EntB0var1-DR	P4	19	**2.3.3** (17), **2.2.1** (1), **2.3.4** (1)
DR1A1-Typhi1-DR-Typhi2-DR-Typhi4-DR-Typhi5-DR15-Typhi6-DR64-DR	DR27-EntB0var1-DR	P5	5	**4.3.1.1** (4), **4.1.0** (1)
DR1A1-Typhi1-DR-Typhi2-DR	DR27-EntB0var1-DR	P6	3	**3.3.1** (3)
DR1A1-Typhi1-DR	DR27-EntB0var1-DR	P7	2	**4.3.1.1** (1), **2.0.0** (1)
DR1A1-Typhi1-DR-Typhi2-DR-Typhi3-DR-Typhi6-DR64-DR	DR27-EntB0var1-DR	P8	2	**4.1.0** (2)
DR1A1-Typhi1-DR-Typhi5-DR15-Typhi6-DR	DR27-EntB0var1-DR	P9	2	**3.0.1** (2)
DR1A1-Typhi1-DR-Typhi2-DR-Typhi3-DR-Typhi4-DR-Typhi5-DR15-Typhi6-DR64-DR64-DR64-DR64-DR64-DR64-DR	DR27-EntB0var1-DR	P10	1	**2.3.4** (1)
DR1A1-Typhi1-DR-Typhi2-DR-Typhi3-DR-Typhi4-DR-Typhi5-DR-Typhi6-DR64-DR	DR27-EntB0var1-DR	P11	1	**4.3.1.1** (1)

## Supplementary Materials

The following are available online at https://www.mdpi.com/article/10.3390/genes12081142/s1. Figure S1: Analysis of various “Group-B” CRISPR loci patterns described by Tanmoy et al. [1]. Table S1: Main characteristics of the 1059 *S. enterica* serovar Typhi genomes studied. Table S2: Discrepancies between genotype data from the study by Tanmoy et al. [1] and our study. Table S3: Strains described in Tanmoy et al. [14] and their different accession codes. Table S4: Correspondence between the spacer and DR sequences described by Tanmoy et al. [1] and those from Fabre et al. [2], and prevalence of these sequences among the 1059 *S. enterica* serovar Typhi genomes studied.Table S5: Correspondence between the “Group-A” CRISPR locus patterns from Tanmoy et al. [1] and our CRISPR profiles.
