# Supplementary material for: Comment on Tanmoy et al. CRISPR-Cas Diversity in Clinical Salmonella enterica Serovar Typhi Isolates from South Asian Countries. Genes 2020, 11, 1365
Source: Genes (Basel). 2021 Jul 28;12(8):1142. doi: 10.3390/genes12081142 (PMC8391509; doi:10.3390/genes12081142)
Supplement: Supplementary file 1 [file genes-12-01142-s001.zip › FigureS1_Fabre_22March2021.pdf]

>pattern b1 (Td23a, Ts55a)

ERR1837236

CGCTTCAGTGGCGAACGTCGTGAAGGCGGTCGTAATTTCAGCGGCGAACGTCGTGAAGGTGGTCGTGGTGATGGTCGTCGCTTCAGTGGCGAACGTCGTGAA

>pattern b9 (Td35a, Td34d, Td35a/Td34d overlapping sequence, SNP)

ERR485140

GTAGACCCTGTTATTCCTGACCCTGTCATTCCCGATCCGGTAGACCCTGATCCAGTAGACCCGGTTATCCCTGACCCTGTTATTCCCGATCCGGTGGACCCTGATCCAGTAGACC
CTGATCCAGTAGACCCGGTCATCCCTGA

> patterns b10-b13 (Td39b (reverse complement of Td39a), Td54a, SNP)

ERR2663897

CACAGGGCTCCAGCGTTGCCGCTTTCCTGAAAAGCCATGAATCACTTTTTGCCAGTACCGCTGTCAGCAGCGCATTCGCAACTCAGAAGCTGG

ERR2663945

TACAGGGCTCCAGCGCTGCCGCTTTCCTGAAAAACCATGAATCACTTCTTGCCGGTACCGCTGTCAGCAGCGCATTCGCAACTCAGAAGCTGGCACAGGGCTCCAGCGCTGCC
GCTTTCCTGAAAAACCATGAATCACTTCTTGCCGGTACCGCTGTCAGCAGCGCATTCGCAACTCAGAAGCTGGCACAGGGCTCCAGCGCTGCCGCTTTCCTGAAAAGCCATGAA
TCACTTTTTGCCAGTACCGCTGTCAGCAGCGCATTCGCAGCTCAGAAGCTGG

ERR2663499

TACAGGGCTCCAGCGCTGCCGCTTTCCTGAAAAACCATGAATCACTTCTTGCCGGTACCGCTGTCAGCAGCGCATTCGCAACTCAGAAGCCGGCACAGGGCTCCAGCGCTGCT
GCTTTCCTGAAAAACCATGAATCACTTCTTGCCGGTACCGCTGTCAGCAGCGCATTCGCAACTCAGAAGCTGGCACAGGGCTCCAGCGCTGCTGCTTTCCTGAAAAACCATGAA
TCACTTCTTGCCGGTACCGCTGTCAGCAGCGCATTCGCAACTCAGAAGCTGGCACAGGGCTCCAGCGCTGCTGCTTTCCTGAAAAACCATGAATCACTTCTTGCCGGTACCGCT
GTCAGCAGCGCATTCGCAACTCAGAAGCTGGCACAGGGCTCCAGCGTTGCCGCTTTCCTGAAAAGCCATGAATCACTTTTTGCCAGTACCGCTGTCAGCAGCGCATTCGCAGCT
CAGAAGCTGG

Figure S1: Analysis of various "Group-B" CRISPR loci patterns described by Tanmoy et al (Genes 2020). The nucleotides of the so-called "DRs" and "spacers" are shown in black and green, respectively. Single nucleotide polymorphisms of either DRs or spacers are indicated in red. The repeated sequences are boxed in color.
